# G2-LIKE CAROTENOID REGULATOR (SlGCR) is a positive regulator of lutein biosynthesis in tomato

**DOI:** 10.1007/s42994-022-00088-z

**Published:** 2022-11-29

**Authors:** Siyan Ren, Yong Yuan, Hsihua Wang, Yang Zhang

**Affiliations:** 1 Key Laboratory of Bio-Resource and Eco-Environment of Ministry of Education, College of Life Sciences, Sichuan University, Chengdu, 610064 China; 2 Sanya Institute of China Agricultural University, Sanya, 572025 China; 3 Beijing Key Laboratory of Growth and Developmental Regulation for Protected Vegetable Crops, College of Horticulture, China Agricultural University, Beijing, 100193 China

**Keywords:** *Solanum lycopersicum*, GARP G2-like transcription factor, Lutein biosynthesis

## Abstract

Lutein is an oxygen-containing carotenoid synthesized in plant chloroplasts and chromoplasts. It plays an indispensable role in promoting plant growth and maintaining eye health in humans. The rate-limiting step of lutein biosynthesis is catalyzed by the lycopene ε-cyclase enzyme (LCYE). Although great progress has been made in the identification of transcription factors involved in the lutein biosynthetic pathway, many systematic molecular mechanisms remain to be elucidated. Here, using co-expression analysis, we identified a gene, *G2-LIKE CAROTENOID REGULATOR* (*SlGCR*), encoding a GARP G2-like transcription factor, as the potential regulator of *SlLCYE* in tomato. Silencing of *SlGCR* reduced the expression of carotenoid biosynthetic genes and the accumulation of carotenoids in tomato leaves. By contrast, overexpression of *SlGCR* in tomato fruit significantly increased the expression of relevant genes and enhanced the accumulation of carotenoids. SlGCR can directly bind to the *SlLCYE* promoter and activate its expression. In addition, we also discovered that expression of *SlGCR* was negatively regulated by the master regulator SlRIN, thereby inhibiting lutein synthesis during tomato fruit ripening. Taken together, we identified SlGCR as a novel regulator involved in tomato lutein biosynthesis, elucidated the regulatory mechanism, and provided a potential tool for tomato lutein metabolic engineering.

**Supplementary Information:**

The online version contains supplementary material available at 10.1007/s42994-022-00088-z.

## Introduction

Carotenoids are a large group of 40-carbon tetraterpenoid pigments that are widely distributed in nature (Liu et al. 2015; Sun et al. 2018). Besides their critical functions in providing distinct colors characterization to flowers, fruits, and vegetables, carotenoids and their derivatives also constitute the vital pigment-protein complexes for photoprotection and light-harvesting that are critically important for plant growth and development (Cao et al. 2019; Niyogi and Truong 2013).

In addition to their primary functions in plants, carotenoids are also essential components of animal diets and human nutrition. Unlike plants, animals and humans cannot themselves synthesize carotenoids and can only obtain them through their diet (DellaPenna and Pogson 2006; Giorio et al. 2013). Indeed, some of these specific carotenoids are essential precursors for vitamin A synthesis, a well-known carotenoid derivative with multi-bioactive functions that are regarded as preventatives of cardiovascular disease and reducing the risk of cancer and other chronic diseases (Fanciullino et al. 2007; Liu et al. 2015; Sandmann et al. 2006).

Lutein is an oxygen-containing carotenoid synthesized in chloroplasts and chromoplasts. In plants, lutein is highly concentrated in the photosynthetic tissues of leaves, due to its primary function as an accessory pigment of the light-harvesting complexes in photosynthesis (DellaPenna and Pogson 2006; Giorio et al. 2013). Besides these functions of participating in the photosynthetic process in green leaves, lutein is also abundant in several vegetative organs, such as fruits and flowers, which has been reported in kiwifruit (Ampomah-Dwamena et al. 2019) and marigold (Fernandez-Sevilla et al. 2010), where it plays an important role in the protection of several triacylglycerols, unsaturated lipids, proteins, and phenol quinones from photooxidation (DellaPenna and Pogson 2006). Although lutein is not categorized as a vitamin, it is considered the most enrichment yellow-colored carotenoid present in the macula lutea, and is vital for maintaining eye health, including the prevention of age-related macular degeneration and as a neuroprotective in the primate retina (Landrum and Bone 2001; Mares 2016). Moreover, during the past decade, increasing medical evidence suggests that lutein plays roles in many biological functions for human health, such as light absorption, reduce oxidative damage, protection against inflammation, and cellular communication to maintain homeostasis (Ahn and Kim 2021; Kijlstra et al. 2012; Liu et al. 2009; Mares 2016).

**Fig. 1:**
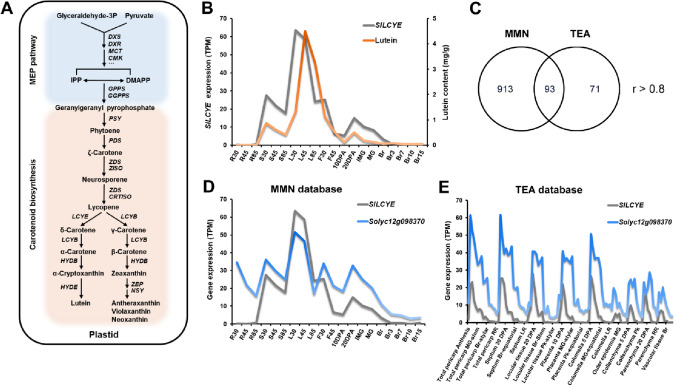
*Solyc12g098370* is highly co-expressed with *SlLCYE* in MicroTom tomato. **A** Schematic representation of the carotenoid biosynthetic pathway. **B** Correlation analysis of lutein content and *SlLCYE* transcript level. **C** The 93 co-expressed candidates shared by both MMN and TEA, with a threshold *r* > 0.8. **D** Co-expression analysis of *SlLCYE* and *Solyc12g098370* transcript levels in the MMN database. Multiple tomato developmental stages of leaves (L), roots (R), stems (S), flowers (F), as well as fruits are shown on the X axis. **E** Co-expression analysis of *SlLCYE* and *Solyc12g098370* transcript levels in the TEA database. Mature green, MG. Red ripe, RR

In plants, lutein biosynthesis begins with the cyclization of lycopene. The lycopene ε-cyclase enzyme (LCYE) catalyzes the rate-limited step to introduce ε-ionone into all-trans lycopene end groups to yield α-carotene (Arango et al. 2014; Sandmann et al. 2006). In addition, both the carotenoid β-ring hydroxylase (HYDB) and carotenoid ε-ring hydroxylase (HYDE) are required to form lutein via a two-step sequential hydroxylation reaction (DellaPenna and Pogson 2006; Isaacson et al. 2002) (Fig. 1A). Previous studies revealed that the expression of *SlLCYE* markedly declined during the tomato fruit ripening process, thus leading to the reduction in lutein content (Klee and Giovannoni 2011; Ronen et al. 1999). The overexpression of *SlLCYE* in tomato fruit resulted in higher lutein content (Wu et al. 2022; Yuan et al. 2022), whereas the knockdown of some genes involved in the carotenoid biosynthetic pathway, including *SlLCYE*, by the CRISPR/Cas9 genome editing technology, resulted in a much lower content of lutein in the mutant tomato fruits (Li et al. 2018b).

Nowadays, great progress has been made in the identification of transcription factors involved in the lutein biosynthetic pathway. A kiwifruit R2R3-MYB transcription factor of *AdMYB7* overexpressed in *N. benthamiana* plants significantly increased the expression of carotenoid biosynthetic genes, including *NbLCYB* and *NbLCYE* (Ampomah-Dwamena et al. 2019). The MYB activator, WP1, interacts with MtTT8 and MtWD40-1 proteins and directly regulates expression of the key genes of *MtLCYE* involved in the lutein biosynthetic pathway (Meng et al. 2019). Moreover, according to transcriptome analysis, the expression of some transcription factors, such as MYB, bHLH, and NAC, were highly correlated with the expression of carotenogenic genes and the content of carotenoid pigments (including lutein) (Li et al. 2018a; Peng et al. 2022), indicating that there are many potential regulators involved in the apocarotenoid accumulation pathway that have not yet been identified. Therefore, elucidating the regulatory mechanism of lutein biosynthesis will extend our understanding of fruit development, as well as provide new strategies for engineering high quality tomatoes.

Under natural selection, expression of genes involved in specialized metabolic pathways sometimes evolved to become highly correlated with one another, temporally and spatially, in plants (Jacobowitz and Weng 2020). Additionally, co-expression analysis can serve as an efficient method for candidate gene identification, across developmental stages, multiple tissue types, or entire biosynthetic pathways (Jacobowitz and Weng 2020; Li et al. 2020a). According to this transcriptome-based weighted gene co-expression network analysis (WGCNA), several transcription factors involved in specialized metabolic pathways have been identified and characterized. For instance, GLYCOALKALOID METABOLISM 9 (GAME9), an APETALA2/ethylene response factor, has been well characterized in regulating steroidal alkaloids biosynthesis in tomato (Cardenas et al. 2016). The CaMYB48 transcription factor, which acts as a transcriptional activator, is involved in capsaicinoid biosynthesis in hot peppers (Sun et al. 2020). In addition, using a WGCNA database, based on all tomato fruit development stages (Shinozaki et al. 2018), a SlWRKY35 transcription factor was identified as a new regulator involved in the carotenoid metabolic pathway in tomato (Yuan et al. 2022). Therefore, co-expression analysis is a powerful tool for predicting gene function that will facilitate the identification of novel potential regulators related to specialized metabolic pathways.

In this study, we used the lutein content during tomato development stages in combination with the MicroTom Metabolic Network (MMN) database (Li et al. 2020a), to identify *G2-LIKE CAROTENOID REGULATOR* (*SlGCR*), which encodes a GARP G2-like transcription factor, as a novel candidate gene in regulating lutein biosynthesis in tomato. Functional studies demonstrated that silencing of *SlGCR* inhibited the expression of lutein biosynthetic genes and reduced the lutein content in tomato leaves. By contrast, the ectopic expression of *SlGCR* in tomato fruit significantly increased the expression of lutein biosynthetic pathway genes, and the content of related carotenoids, as well as lutein. Further tests revealed that SlGCR exerts a direct transcriptional activation on the *SlLCYE* promoter, encoding a key rate-limiting enzyme involved in the lutein biosynthetic pathway. Finally, based on these findings, a model is proposed in which *SlGCR* is negatively regulated by SlRIN, during tomato fruit ripening, which may provide new insights into the ripening-related reduction of lutein content in the tomato fruit.

## Results

### *Solyc12g098370* is a candidate gene involved in the transcriptional regulation of *SlLCYE*

To identify novel transcription factors involved in the regulation of lutein metabolism, we first determined the lutein contents in tomato tissues, at different developmental stages (Supplemental Fig. 1). The lycopene ε-cyclase, encoded by *LCYE*, introduces a single ε-ring into lycopene to finally produce lutein, serves as a key rate-limiting enzyme in lutein biosynthesis (Cunningham et al. 1996). Based on the lutein content and using the MicroTom Metabolic Network (MMN) database (Li et al. 2020a), we observed a high correlation between the expression levels of *SlLCYE* (*Solyc12g008980*) and lutein (Fig. 1B). This finding indicated that lutein accumulation is significantly affected by the expression of *SlLCYE* transcription in MicroTom tomato. Therefore, we began to focus on the transcriptional regulation of *SlLCYE*.

**Fig. 2:**
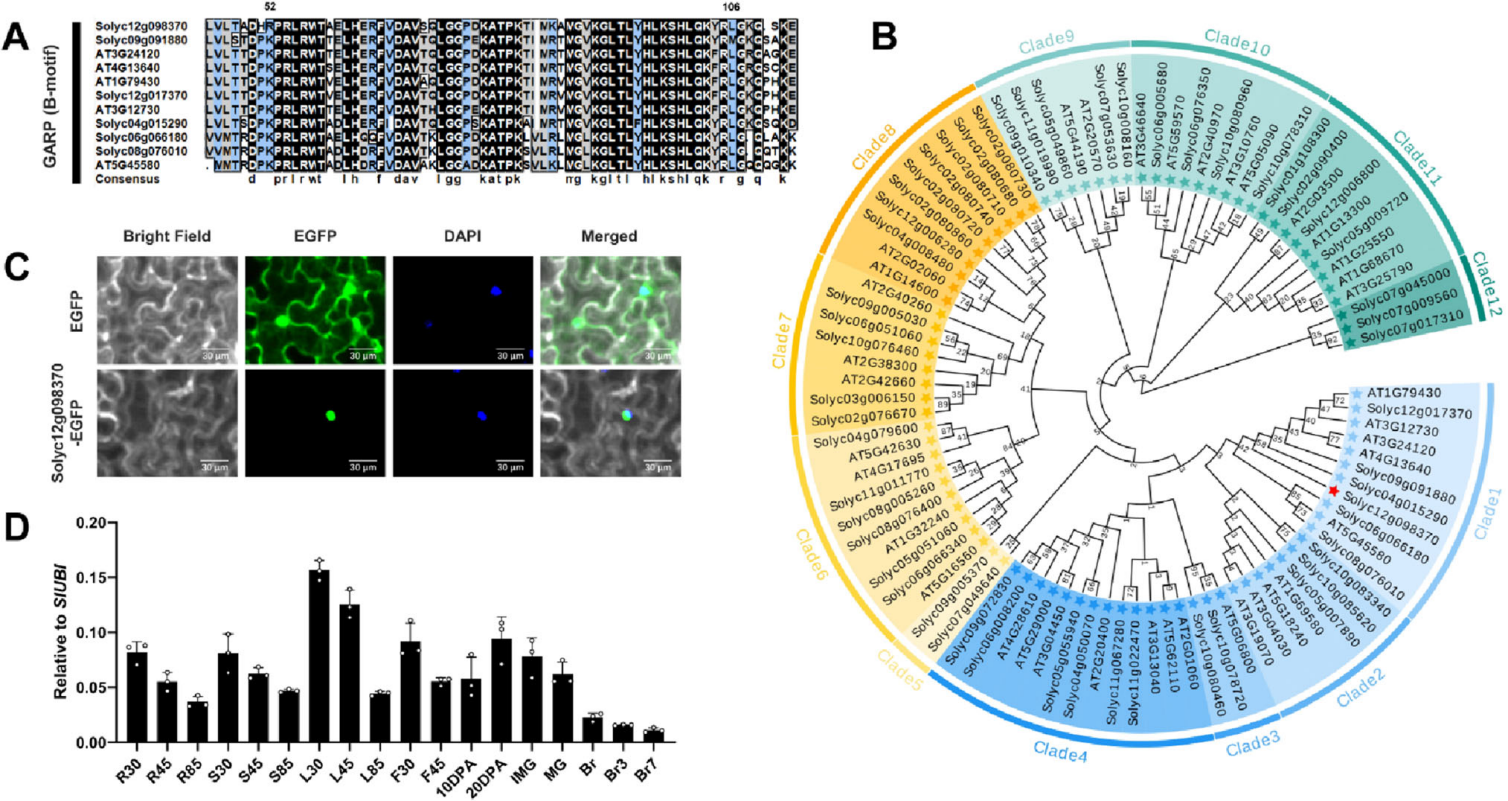
*Solyc12g098370* encodes a GARP G2-like transcription factor. **A** Multi-sequence alignment of the B motif. **B** Phylogenetic analysis of SlG2-likes and AtG2-likes. Solyc12g098370 is represented by a red star in clade 1. **C** Subcellular location of the Solyc12g098370 protein. DAPI staining serves as the nuclear marker. Scale bars, 20 μm. **D** RT-qPCR analysis showing the tissue-specific expression pattern of *Solyc12g098370* in MicroTom. Root (R), stem (S), leaf (L), and flower (F) samples were harvested at 30 DPG, 45 DPG, and 85 DPG. Fruit samples were harvested at 10 DPA, 20 DPA, immature green (IMG), mature green (MG), breaker (Br), 3 days post breaker stage (Br3), and 7 days post breaker stage (Br7). Error bars represent the SD (*n* = 3)

Using the transcript levels of *SlLCYE* as a bait, co-expression analysis was carried out in the MMN database and the Tomato Expression Atlas database (TEA, http://tea.solgenomics.net/) (Shinozaki et al. 2018), respectively, to screen for co-expressed genes encoding transcription factors (threshold *r* > 0.8). Of the 93 co-expressed candidates shared by both MMN and TEA, *Solyc12g098370* was the only gene encoding a transcription factor (Fig. 1C–E). Multi-sequence alignment showed that Solyc12g098370 had a highly conserved B motif, which is the signature domain of the GARP family members (Safi et al. 2017) (Fig. 2A). The GARP family is a plant-specific transcription factor family, consisting of G2-like and ARR-B subclasses (Safi et al. 2017). Further phylogenetic analysis showed that Solyc12g098370 belongs to the G2-like subfamily (Fig. 2B).

The full length of the open reading frame (ORF) of *Solyc12g098370* was cloned for subcellular localization assays. The recombinant vector was introduced into the *Nicotiana benthamiana* leaves, via *Agrobacterium tumefaciens* infiltration. An empty EGFP vector was used as a control. Fluorescence microscopy showed that the green fluorescent signal for the control was observed primarily in the cytoplasm, whereas the EGFP fusion protein signal was only detected in the nucleus, demonstrating that Solyc12g098370 is a nuclear protein (Fig. 2C).

The expression levels of *SlGCR* in different tomato developmental stages and tissues were determined by reverse transcription-quantitative PCR (RT-qPCR) (Fig. 2D). *SlGCR* is ubiquitously expressed at different developmental stages in MicroTom. The expression levels of *Solyc12g098370* were similar in roots, stems and flowers, but markedly higher in leaves. In fruits, the expression levels of *SlGCR* decreased sharply during fruit ripening and subsequently remained at a low level. In summary, the expression levels of *Solyc12g098370* were much higher in tomato green tissues, but extremely low in mature fruits, exhibiting a spatio-temporal specific expression pattern.

### Silencing of *Solyc12g098370* inhibits carotenoid biosynthesis in tomato leaves

**Fig. 3:**
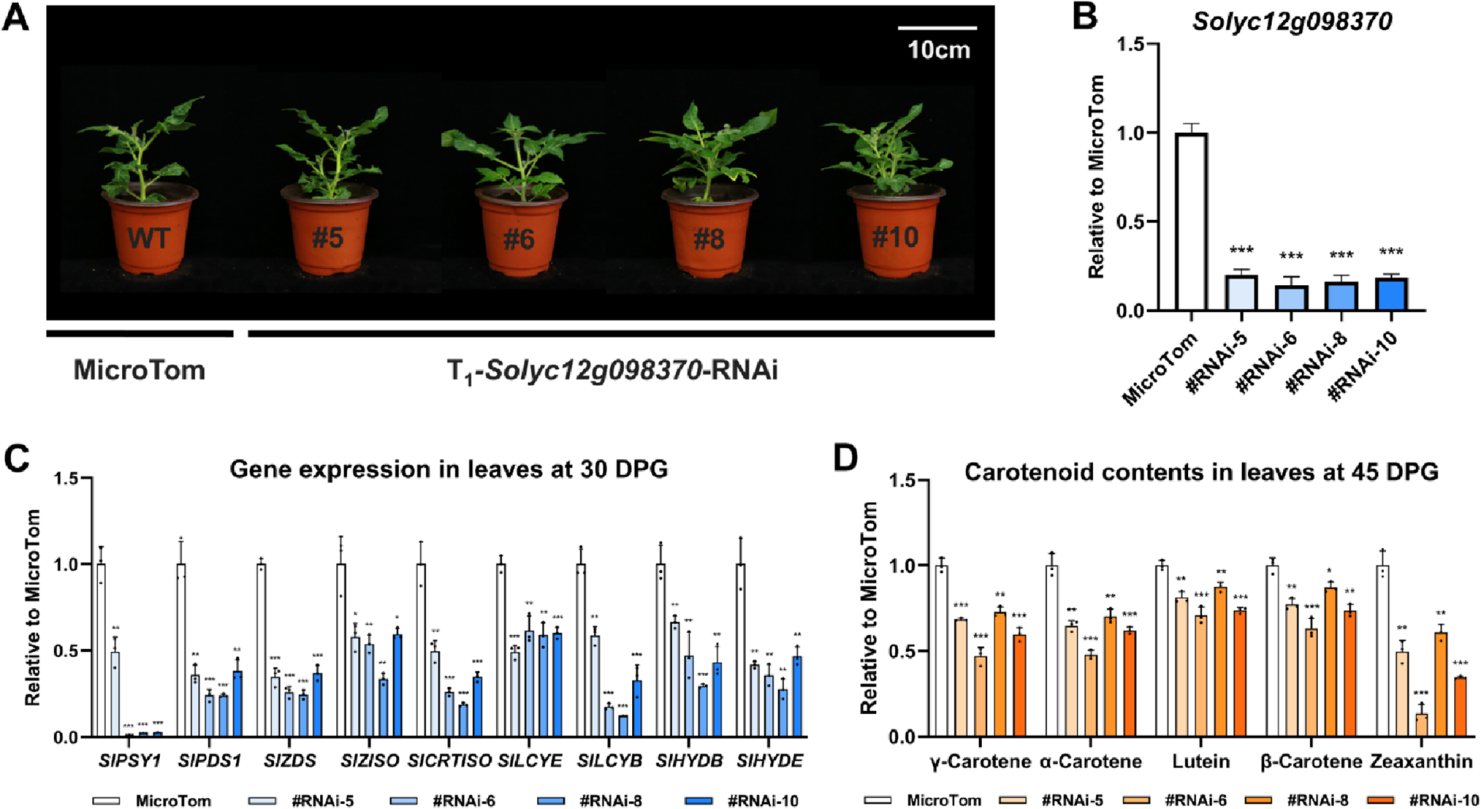
The carotenoid biosynthesis is inhibited in the leaves of *Solyc12g098370-*RNAi lines. **A** Photograph of MicroTom and T_1_-*Solyc12g098370*-RNAi at the 30 DPG stage. Scale bar, 10 cm. **B** RT-qPCR analysis shows that the expression levels of *Solyc12g098370* were significantly decreased in the leaves of RNAi lines. **C** RT-qPCR analysis showing the expression levels of carotenoid biosynthetic genes in leaves at the 30 DPG stage relative to MicroTom. **D** Contents of the major carotenoids in leaves at the 45 DPG stage relative to MicroTom. Error bars represent the SD (*n* = 3). *(*P* < 0.05), **(*P* < 0.01) and ***(*P* < 0.001) compared to MicroTom at the same stage (Student’s *t*-test)

To determine whether *Solyc12g098370* is involved in tomato lutein biosynthesis, the *Solyc12g098370*-RNAi vector was constructed and transferred to the MicroTom background to generate transgenic plants with a reduced expression level of *Solyc12g098370*. The tissue expression pattern showed that *Solyc12g098370* maintained at a high, and relatively stable, expression level in young wild-type tomato leaves (Fig. 2D). Therefore, we considered leaves to be the best tissue for detecting the effects of gene silencing. Though there were no significant differences in phenotypes between transgenic lines and wild type plants (Fig. 3A), RT-qPCR analysis indicated that the *Solyc12g098370* expression levels were significantly decreased in leaves of the *Solyc12g098370*-RNAi lines (Fig. 3B). Consistently, expression levels of the carotenoid biosynthetic genes, *SlPSY1*, *SlPDS1*, *SlZDS*, *SlZISO*, *SlCRTISO*, *SlLCYE*, *SlLCYB*, *SlHYDB*, *SlHYDE*, were all decreased compared to those in the MicroTom control plants (Fig. 3C). Consistent with these findings, our analytical assays showed that the contents of major carotenoids, including γ-carotene, α-carotene, lutein, β-carotene, and zeaxanthin, were all significantly reduced compared with MicroTom leaves (Fig. 3D and Table S1). Together, these findings suggest that *Solyc12g098370* is involved in the metabolism of tomato carotenoids, including lutein. Therefore, we renamed this *Solyc12g098370* gene as *G2-LIKE CAROTENOID REGULATOR* (*SlGCR*).

### Fruit-specific overexpression of *SlGCR* enhances lutein biosynthesis in tomato fruit

To further confirm the role of *SlGCR* in the regulation of carotenoid biosynthesis in the tomato fruit, the fruit-specific *E8* promoter was selected to drive the overexpression of *SlGCR* (Supplemental Fig. 2A). Among 10 independent positive transgenic plants obtained in the T_0_ generation, two lines with high *SlGCR* expression, *E8:SlGCR*-5, and *E8:SlGCR*-6, were selected for further analysis (Supplemental Fig. 2B, C).

**Fig. 4:**
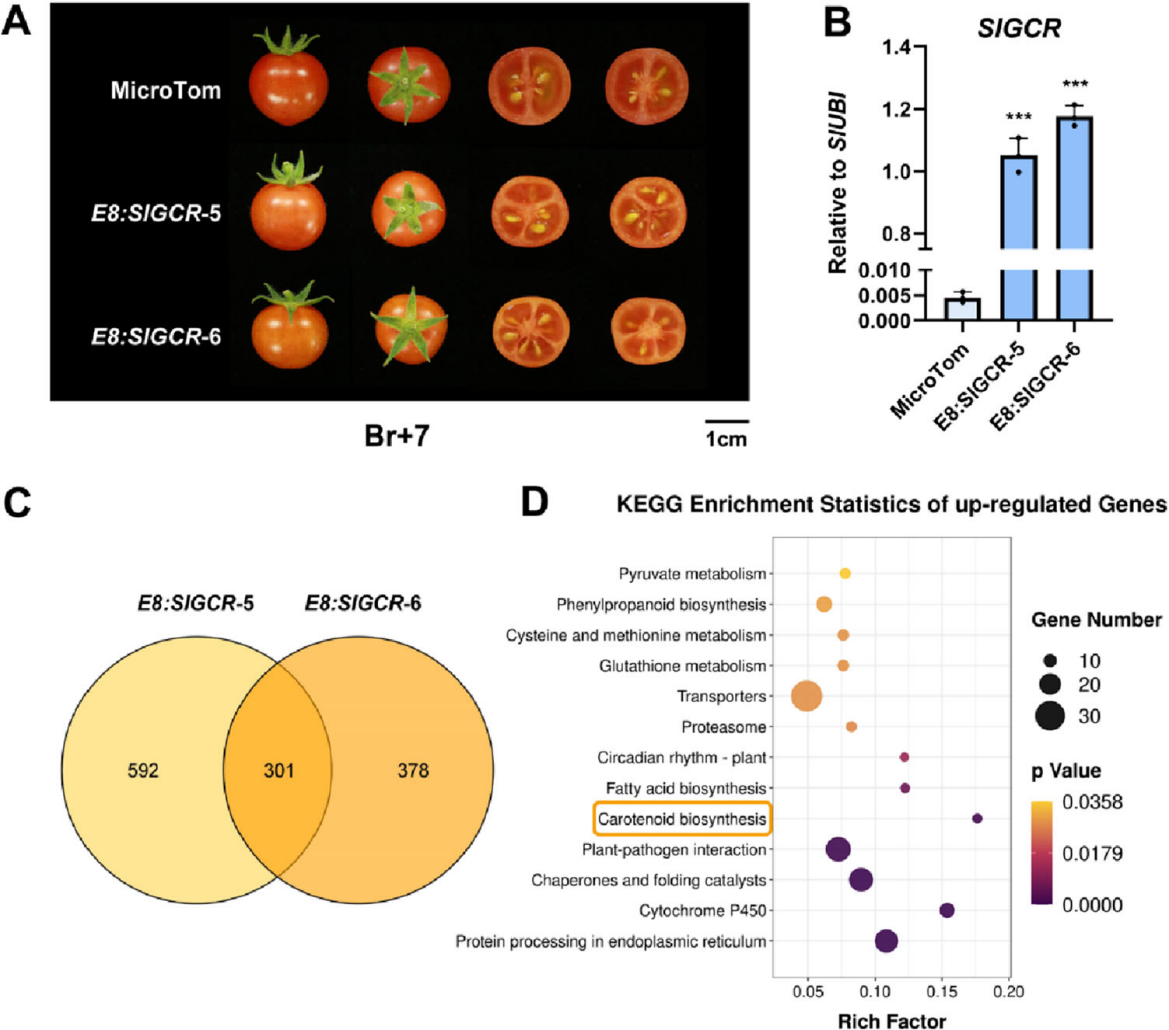
Fruit-specific overexpression of *SlGCR* promotes carotenoid accumulation in tomato fruits. **A** T_1_-*E8:SlGCR* fruits showing an orange phenotype. **B** RT-qPCR analysis showing the expression levels of *SlGCR* in T_1_-*E8:SlGCR* fruits at the Br7 stage. **C** Venn diagram showing the common and specific upregulated genes in T_1_-*E8:SlGCR*-5 and 6. **D** KEGG enrichment statistics of co-upregulated genes in both T_1_-*E8:SlGCR* lines. Rich factor reflects the proportion of differentially expressed genes in a given pathway. The size of each node represents the number of enriched genes. *P* values are indicated by different colors, changing from yellow to purple

**Fig. 5:**
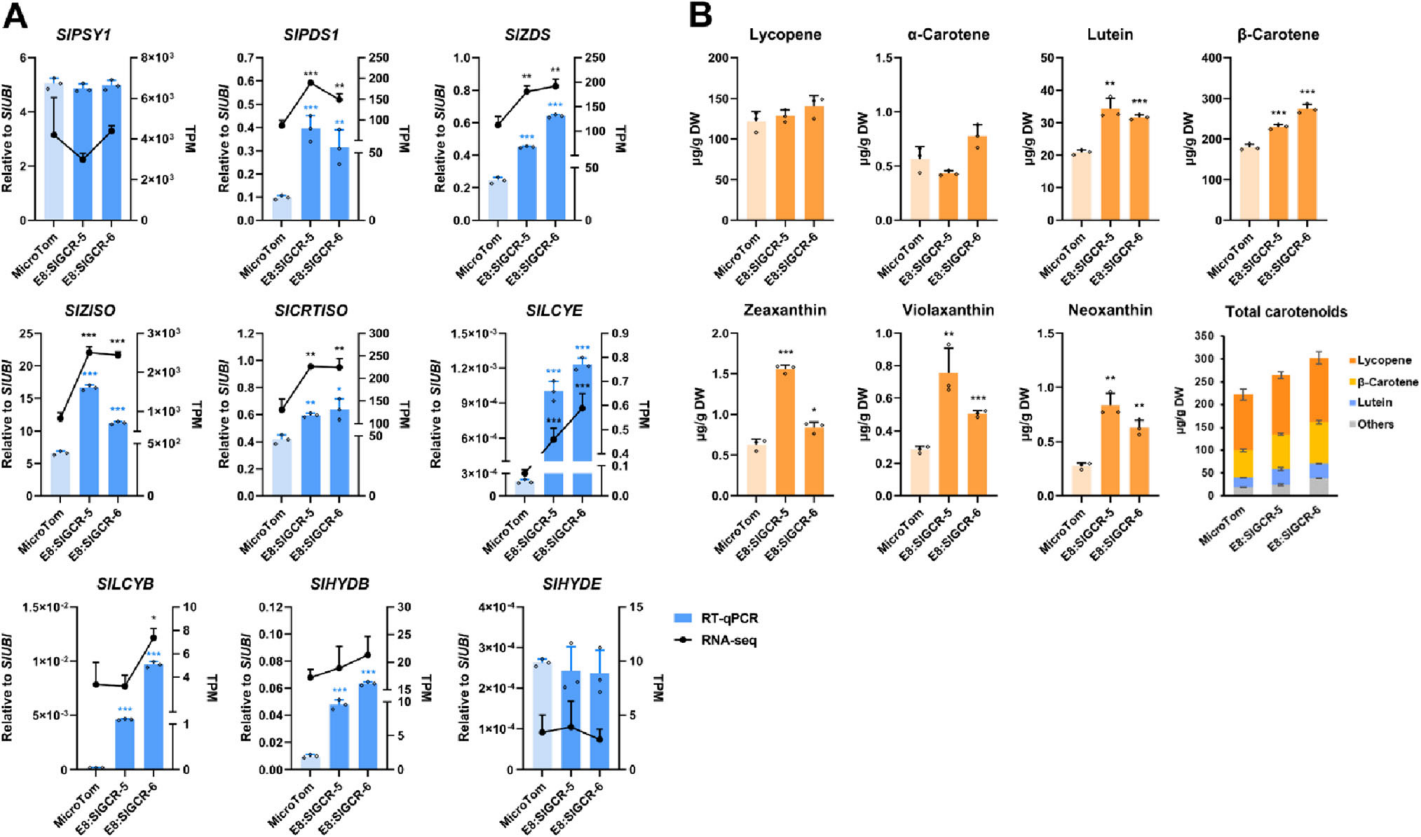
Overexpression of *SlGCR* in tomato fruit activates the carotenoid pathway. **A** The RNA-seq and RT-qPCR analysis showing the transcript levels of carotenoid biosynthetic genes in tomato fruits at the Br7 stage. **B** Contents of the major carotenoids in tomato fruits at the Br10 stage. Error bars represent the SD (*n* = 3). *(*P* < 0.05), **(*P* < 0.01) and ***(*P* < 0.001) compared to MicroTom at the same stage (Student’s *t*-test)

Compared to the red pericarp of MicroTom, fruits of both *E8:SlGCR* tomato lines showed orange phenotypes at the Br7 stage, indicating significant changes in metabolites (Fig. 4A, B). RNA-sequencing (RNA-seq) analysis was subsequently performed on the fruits (Br7) of *E8:SlGCR* transgenic lines and MicroTom, to further elucidate the molecular mechanism underlying the phenotypic changes caused by *SlGCR* overexpression. Here, we identified some 301 upregulated and 257 downregulated differentially expressed genes (DEGs), shared by both lines, compared with MicroTom (Fig. 4C and Supplemental Fig. 3). KEGG enrichment analysis showed that many upregulated genes were enriched in several metabolic pathways, including carotenoid biosynthesis (Fig. 4D). Consistently, RNA-seq and RT-qPCR results showed that the expression levels of most carotenoid biosynthetic genes were significantly higher than those of MicroTom (*SlPDS1*, *SlZDS*, *SlZISO*, *SlCRTISO*, *SlLCYE*) (Fig. 5A).

The results from our analytical assays showed that the lycopene and α-carotene contents were not significantly changed, whereas the accumulation levels of lutein, β-carotene, zeaxanthin, violaxanthin, neoxanthin and other downstream products were significantly increased, leading to a considerable increase in the total carotenoid contents in tomato fruits, at the Br10 stage (Fig. 5B). Taken together, these results supported the hypothesis that SlGCR is a positive regulator of carotenoid biosynthesis.

### SlGCR binds directly to the promoter of *SlLCYE* and activates its expression

The findings from our RT-qPCR analyses established that expression levels of the carotenoid biosynthetic genes were significantly decreased in the leaves of the RNAi lines (Fig. 3C). Among which, the expression levels of seven genes (*SlZDS*, *SlZISO*, *SlCRTISO*, *SlLCYE*, *SlLCYB* and *SlHYDB*) were significantly increased in the fruits of overexpression lines (Supplemental Fig. 4A), indicating that they might be the direct targets of SlGCR.

**Fig. 6:**
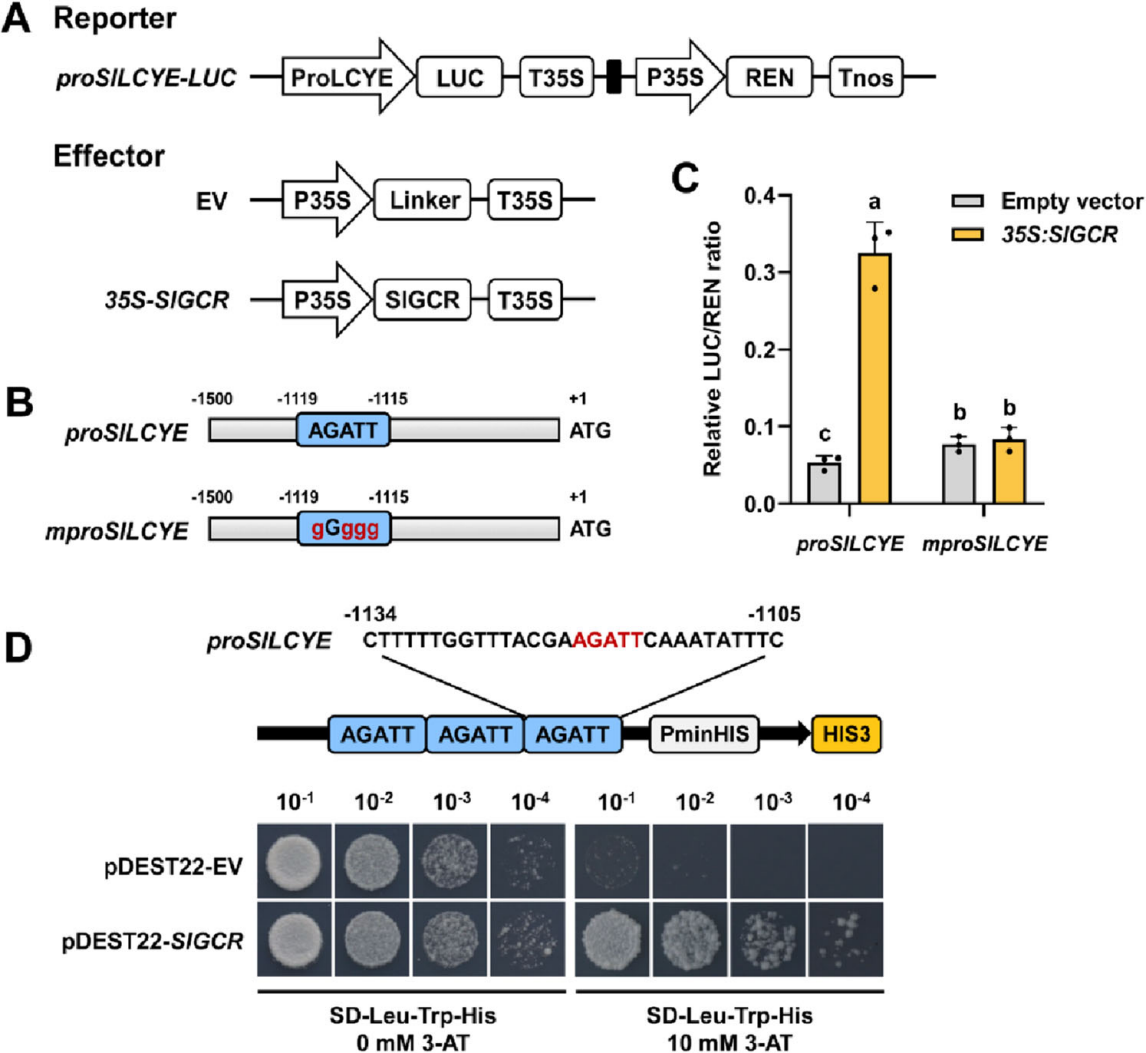
SlGCR directly binds to the *SlLCYE* promoter to activate its expression. **A** Schematic diagrams of vectors used for Dual-Luciferase assay. **B** Schematic diagrams of mutant *SlLCYE* promoter used as a negative control. **C** Relative LUC/REN ratio showing the significantly promoted transcriptional activity of *SlLCYE* promoter caused by SlGCR, while no significance exists in the mutant group. Error bars represent the SD (*n* = 3). Different letters above the error bars indicate significant differences (*P* < 0.05, Student’s *t*-test). **D** Yeast-one-hybrid (Y1H) assay showing that SlGCR binds directly to the AGATT elements on the *SlLCYE* promoter. The AGATT element of the *SlLCYE* promoter was repeated three times and fused to the upstream of the *His3* reporter gene. SD medium minus leucine, tryptophan and histidine, SD-Leu-Trp-His. 3-amino-1,2,4-triazole, 3-AT

The B-motif, which is the signature domain of the GARP family, can bind specifically to the AGATT cis-acting elements to regulate target genes (Fitter et al. 2002; Hosoda et al. 2002). Therefore, we next examined the promoter sequences (1500 bp upstream of the ATG site) of these potential target genes and established that the AGATT elements were present in the promoters of *SlPSY1, SlZDS*, *SlZISO*, *SlCRTISO*, *SlLCYE*, *SlLCYB SlHYDB* and *SlHYDE* (Supplemental Fig. 4B). These promoters were then cloned from the MicroTom genome and used in dual-luciferase reporter assays to verify whether they could be directly activated by SlGCR (Fig. 6A). These assays showed that the *SlLCYE* promoter was significantly activated by SlGCR in *N. benthamiana* leaves, whereas the other promoters were not activated by SlGCR (Fig. 6C and Supplemental Fig. 4C). Here, it is noteworthy that this activation was removed after the AGATT element of the *SlLCYE* promoter was mutated (Fig. 6B, C).

To verify a direct interaction between SlGCR and the *SlLCYE* promoter, we employed a yeast one-hybrid (Y1H) system. These results showed that the yeast cells could grow on the SD-/Leu-/Trp-/His medium supplemented with 10 mM 3-amino-1,2,4-triazole (3-AT) when co-transferred with *pHIS-LEU2-proSlLCYE* and pDEST™22-*SlGCR*, but the control group could not survive on the same medium, indicating that SlGCR can directly bind to the AGATT cis-acting element on *SlLCYE* promoter (Fig. 6D). Together, these findings provided support for our model in which SlGCR binds directly to the *SlLCYE* promoter to activate its expression.

### The expression of *SlGCR* is negatively regulated by SlRIN

**Fig. 7:**
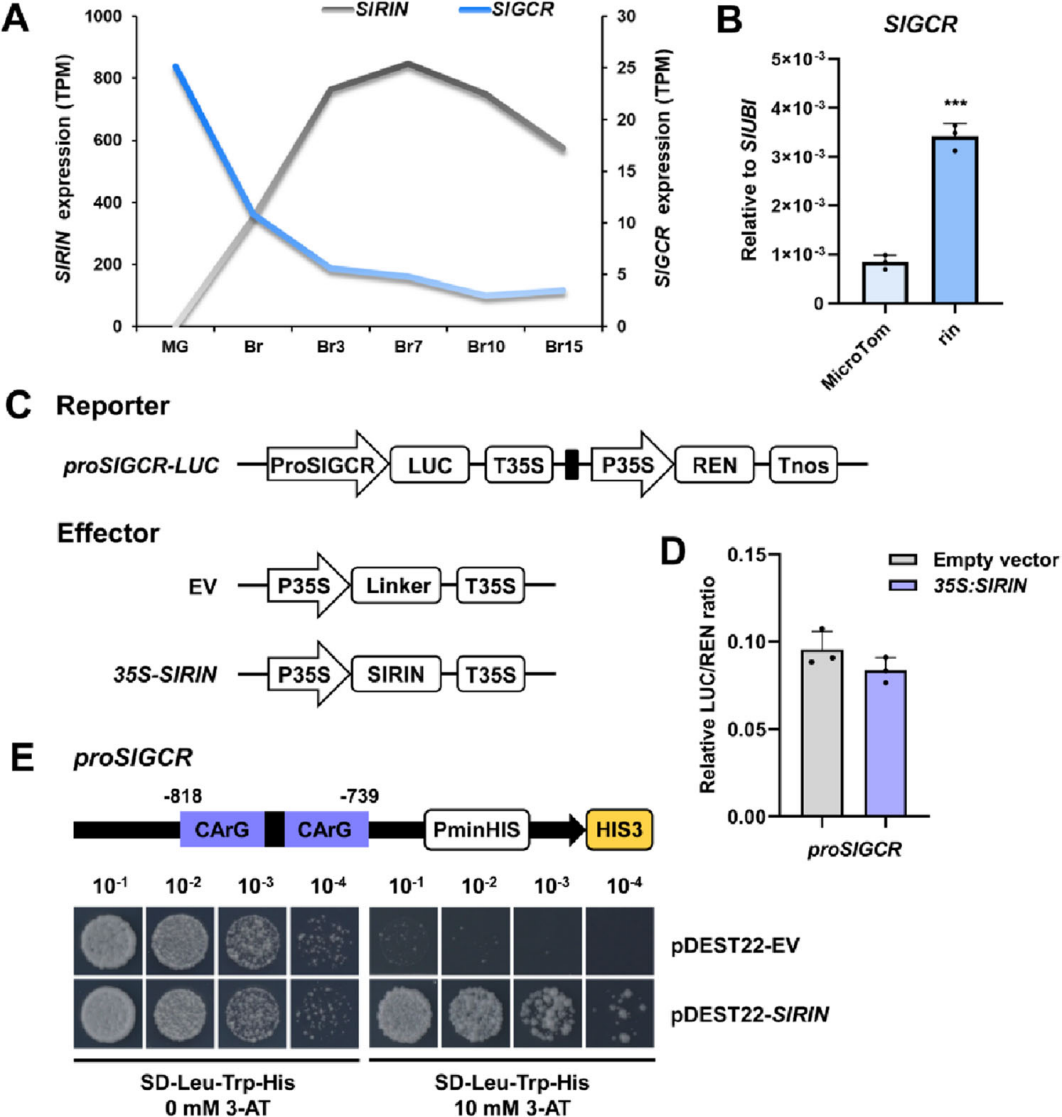
*SlGCR* is negatively regulated by ripening regulator SlRIN**. A** Co-expression analysis shows an opposite expression pattern between *SlLCYE* and *SlGCR* in the MMN database. **B** RT-qPCR analysis showing the expression level of *SlGCR* in *rin* mutant fruits at the Br15 stage. Error bars represent the SD (*n* = 3). ***(*P* < 0.001) compared to MicroTom at the same stage (Student’s *t*-test). **C** Schematic diagrams of vectors used for Dual-Luciferase assay. **D** Relative LUC/REN ratio showing that SlRIN had no significant effect on the transcriptional activity of *SlGCR* promoter. **E** Y1H assay showing that SlRIN directly binds to the CArG boxes on the *SGCR* promoter. The region of the *SlGCR* promoter containing 2 CArG boxes was cloned and fused to the upstream of the *His3* reporter gene. SD medium minus leucine, tryptophan and histidine, SD-Leu-Trp-His. 3-amino-1,2,4-triazole, 3-AT

To explore the underlying basis for the reduction of *SlGCR* expression, during tomato fruit ripening, we performed a co-expression analysis of *SlGCR* transcript levels in the MMN database to screen for genes that were negatively correlated with *SlGCR*. Here, we identified *SlRIN*, encoding a master regulator of fruit ripening in tomato (Ito et al. 2017; Vrebalov et al. 2002), as it exhibited an opposite expression pattern to *SlGCR* in the fruits of MicroTom (Fig. 7A). RT-qPCR analysis showed that *SlGCR* expression level was significantly increased in the fruits of the *rin* mutant (Ito et al. 2017), compared to that in MicroTom (Fig. 7B). Therefore, we speculated that *SlGCR* is negatively regulated by *SlRIN* in fruit ripening.

An earlier chromatin immunoprecipitation sequencing (ChIP-seq) study revealed the enrichment of SlRIN on the *SlGCR* promoter (Zhong et al. 2013) (Supplemental Fig. 5A). In addition, two CArG boxes, which were previously reported to be the direct target of SlRIN (Ito et al. 2008; Nakano et al. 2011), were found in the *SlGCR* promoter (Supplemental Fig. 5B). The results of Y1H assays further confirmed the direct interaction between SlRIN and the *SlGCR* promoter (Fig. 7E). However, results from the dual-luciferase reporter assays showed that SlRIN had no direct effect on the transcriptional activity of the *SlGCR* promoter. (Fig. 7C, D). These findings are consistent with a model in which *SlGCR* is negatively regulated by SlRIN, through its direct binding to the CArG boxes in the *SlGCR* promoter. However, as SIRIN cannot directly inhibit *SlGCR* expression, this likely reflects the involvement of a more complex regulatory mechanism.

## Discussion

In plants, carotenoids, especially xanthophylls, play key roles in protecting the photosynthetic systems against photooxidative damage (Havaux and Niyogi 1999). Lutein is one of the most abundant xanthophylls in photosynthetic organisms that constitute the essential components of the light-harvesting complexes (DellaPenna and Pogson 2006; Holt et al. 2005). Throughout the tomato developmental stages, lutein was highly accumulated in photosynthetic tissues, for example, in the leaves, and was sharply decreased to an almost undetectable level in the ripening fruit (Fig. 1B). Based on correlation analysis of the lutein content and the expression pattern of *SlLCYE*, we characterized *SlGCR* as a novel transcription factor that directly activates the expression of *SlLCYE*, the key rate-limiting enzyme gene involved in the lutein metabolic pathway, thus consequently inducing downstream lutein biosynthesis.

In the present study, silencing of *SlGCR* reduced expression of the carotenoid biosynthetic genes as well as the accumulation of carotenoids in tomato leaves (Fig. 3). The overexpression of *SlGCR* in tomato fruit activated the expression of carotenoid biosynthetic pathway genes, including *SlLCYE*, thus leading to a considerable increase of carotenoids (Fig. 5). However, even though we observed a significant increase in lutein within the *SlGCR* overexpressed tomato fruit, this degree of improvement was far from the level achieved by overexpression of *SlLCYE* alone (Yuan et al. 2022). This might be explained by SlGCR serving as a comprehensive activator that can simultaneously activate the expression of genes involved in the carotenoid biosynthetic pathway, such as *SlLCYB*, which directs the metabolic flux downstream of lycopene towards the pathway for the production of β-carotene, thus we detected a significant increase of β-carotene, zeaxanthin, violaxanthin, and neoxanthin in the *E8:SlGCR* overexpression tomato fruits (Fig. 5). On the other hand, synthesis of lutein in tomato fruit is also hampered by a tightly regulated physiological mechanism and the degradation of chloroplasts that occurs before the start of ripening, to a large extent determines the upper limit of lutein synthesis (Giorio et al. 2013; Stigliani et al. 2011).

Tomato fruit ripening consists of a complex network, has been well characterized in an ethylene-dependent manner and great progress has been made in the identification of transcription factors responsible for that climacteric process. The MADS-RIN (SlRIN) transcription factor has long been considered to function as a master regulator that is essential for the induction of tomato fruit ripening, which is accompanied by the accumulation of carotenoids, cell wall softening, acids and sugars metabolism, and production of multiple aroma volatiles (Li et al. 2020b; Vrebalov et al. 2002). This suggests that many other transcription factors may also contribute to the regulation of these processes, directly, or via the forming of heterodimers. SlNAC4 protein can interact with both RIN and NOR transcription factors for affecting ethylene synthesis and carotenoid accumulation (Zhu et al. 2014). SlRIN has been shown to interact with a MADS protein, TAGL1, to form heterodimers to regulate the expression of tomato fruit cell wall softening genes (Li et al. 2019). Yeast one-hybrid assays confirmed that SlRIN protein can directly interact with the CArG-motif in the *SlGCR* promoter (Fig. 7E). However, we failed to observe a direct inhibition of the transcriptional activity of the *SlGCR* promoter, based on our dual-luciferase reporter assays (Fig. 7D), consistent with the notion that there might be a more complex regulatory mechanism for *SlGCR* transcriptional activation. Evidence consistent with such complexity is offered by the finding that the LONG HYPOCOTYL 5 (HY5) and PHYTOCHROME INTERACTING FACTOR 1 (PIF1) form a dynamic activation-suppression transcriptional module that antagonistically regulates carotenoid accumulation in plants (Meng et al. 2019; Toledo-Ortiz et al. 2014).

Tomato is an ideal crop for engineering high-value carotenoid derivatives (Li et al. 2018b, c), due to the natural accumulation of lycopene in the ripe fruit. This property has allowed tomato to serve as the essential precursor for plenty of apocarotenoid biosynthesis with biological properties, including astaxanthin (Huang et al. 2013), crocin (Ahrazem et al. 2022), zeaxanthin and lutein (Giorio et al. 2013; Wu et al. 2022). Lutein has been proven to have an important protective effect on the retina of the human eye, and there is a present expansion of health products based on using lutein as a food additive (Giorio et al. 2013). However, most of the current production of lutein is derived from using the petals of marigold flowers (*Tagetes erecta*), which its always accompanied by low productivity, labor-intensive, and high production costs of this system (Fernandez-Sevilla et al. 2010). Given this situation, more efficient agricultural systems or different biofortified species are required to satisfy these growing market demands. In our previous study, we successfully engineered high-value metabolites in tomato fruit, such as flavonoids (Zhang et al. 2015), glycoalkaloids (Li et al. 2020a), and carotenoids (Yuan et al. 2022). Hence, SlGCR could be used as a new regulator and useful tool for engineering lutein biosynthesis in tomato, which has the potential to be developed for the production of food additives, feed, or other useful carotenoid by-products.

## Materials and methods

### Plant materials and growth conditions

Tomato (*Solanum lycopersicum* cv. MicroTom) seeds were purchased from PanAmerican seed. MicroTom plants were grown in a standard greenhouse at 24 °C for 16 h during the day and 8 h during the night cycles with 60% humidity and 250 μmol m^−2^ s^−1^ light intensity. Roots, stems, leaves, flowers, and fruit pericarps were harvested at several stages, immediately frozen in liquid nitrogen and stored at − 80 °C for further investigation. The *Nicotiana benthamiana* seedlings used for subcellular location and dual-luciferase reporter assays were grown in the above-described conditions.

### Co-expression analysis

Using the transcript levels of *SlLCYE* as a bait, co-expression analysis was carried out in the MicroTom Metabolic Network (MMN) database (Li et al. 2020a) and the Tomato Expression Atlas database (TEA, http://tea.solgenomics.net/), respectively, to screen for co-expressed genes encoding transcription factors (threshold *r* > 0.8). The co-expressed genes shared by both databases were then selected for further analysis.

### Phylogenetic analysis

The protein sequences of AtG2-likes and SlG2-likes were downloaded from the Plant Transcription Factor Database (PlantTFDB, http://planttfdb.gao-lab.org/). Multiple sequence alignments were then performed, using the ClustalW algorithm in MEGA 7.0 (https://www.megasoftware.net/), with the default parameters. The alignment results were subsequently used to construct a phylogenetic tree, using the neighbor-joining method with 1000 bootstrap replicates in MEGA 7.0. The phylogenetic tree was displayed with EvolView (https://evolgenius.info/).

### Construction of plasmids and generation of transgenetic plants

For the RNAi vector, the specific DNA fragment of *SlGCR* (200 bp) was first cloned into the pDONR207 entry vector (Mohanty et al. 2008) and subsequently assembled into the destination vector, pHellsgate12, using the Gateway Cloning Technology (Curtis and Grossniklaus 2003). With the GoldenBraid system (Sarrion-Perdigones et al. 2013), the full-length coding sequence (CDS) of *SlGCR* was used to generate a fruit-specific expression vector, under the control of *E8* promoter, in addition to a kanamycin resistance gene driven by *NOS* promoter. These plasmids were transformed to *S. lycopersicum* via *Agrobacterium tumefaciens*, as described previously (McCormick et al. 1986).

### RNA-seq and RT-qPCR analysis

Fruit pericarps of MicroTom and transgenic lines were harvested at the Br7 stage for use of both RNA-sequencing (RNA-seq) and reverse transcription-quantitative PCR (RT-qPCR) analyses. RNA-seq was performed at Beijing Novogene Bioinformatics Technology Co., Ltd, via the Illumina HiSeq X Ten platform. Clean reads were mapped to the tomato reference genome (Tomato Genome Consortium 2012), and then normalized to transcripts per million (TPM). Differentialy expressed genes (DEGs) were identified by a significance threshold of log2-fold-change of ± 1. All raw sequence data have been deposited in the Genome Sequence Archive at the Big Data Center, Beijing Institute of Genomics, Chinese Academy of Sciences, under the accession number CRA007919, which is publicly accessible at https://ngdc.cncb.ac.cn/gsa (Chen et al. 2021; Memberspartners 2022).

Total RNA was extracted from each sample using the RNeasy Mini Kit (Qiagen, Stockach, Germany). The PrimeScript RT Reagent Kit (Takara Bio, Kusatsu, Japan) was used for genome removal and reverse transcription reaction. The cDNA obtained was then used as a template for RT-qPCR, via the Bio-Rad CFX384 Real-Time System. *SlUBI* (*Solyc01g056940*) was used as an endogenous reference gene to calculate the relative expression levels of target genes. Primers used for RT-qPCR were designed by the qPCR Primer Database (https://biodb.swu.edu.cn/qprimerdb/) and are shown in Supplemental Table S2.

### Extraction and determination of carotenoids

For carotenoid extraction, leaves were harvested at the 45 DPG stage, and fruit pericarps were harvested at the Br10 stage. Fresh tissues were then frozen in liquid nitrogen immediately and then lyophilized. The extraction steps were modified from the previous method (Petry and Mercadante 2018). 50 mg of lyophilized powder was dissolved in 500 μL pre-mixed solution of n-hexane: methanol: acetone (2:1:1, V/V/V), then vortex mixed and sonicated for 20 min at room temperature. After centrifugation for 5 min, the supernatant was collected, concentrated in a vacuum centrifugal concentrator, and then redissolved in 1 mL methanol, followed by sonication at 4 °C for 5 min. After centrifugation for 10 min, the supernatant was passed through a 0.22 μm filter before determination. The product analysis was performed on an Ultimate3000 Series UPLC (Thermo Scientific, MA, USA) and an Accucore C30 column (Thermo Scientific, MA, USA). The column temperature was set at 20 °C. 100% acetonitrile was used as the mobile phase A, methyl tert-butyl ether was used as the mobile phase B, and ultrapure water was used as the mobile phase C. The mobile gradient was as follows: 0–1 min, 90% A and 10% C; 1–2 min, 100% A; 2–4.5 min, 85% A and 15% B; 4.5–7.5 min, 100% A; 7.5–10 min, 90% A and 10% C. The flow rate was 1 mL/min and the injection volume was 2 μL. Detection was performed at 450 nm. Carotenoid standards were purchased from Sigma (https://www.sigmaaldrich.cn/CN/zh), as previously described (Wu et al. 2022). The Chromeleon7.2 SR4 software was used for data analysis.

### Dual-luciferase reporter assay

The 1500-bp promoter regions upstream of the ATG site of *SlLCYE* and *SlGCR* were amplified by PCR and cloned into the pUPD2 entry vector to generate the reporter constructs, via the GoldenBraid system (Sarrion-Perdigones et al. 2013). Full-length CDS of *SlGCR* and *SlRIN* were assembled into the pEAQ-HT-DEST2 vector using the Gateway Cloning Technology to generate the effector constructs (Curtis and Grossniklaus 2003). An empty pEAQ-HT-DEST2 vector was used as a control. The recombinant vectors were transformed into *A. tumefaciens* strain GV3101. *A. tumefaciens* cultures expressing reporters and effectors were mixed in equal proportions and then infiltrated into 3–4 weeks old *N. benthamiana* leaves, as previously described (Niu et al. 2020). LUC and REN activities were measured using the Dual-Luciferase Reporter Assay System (Promega, Madison, USA) following the manufacturer’s instructions. Relative LUC/REN ratios were calculated, representing the transcriptional activity of promoters.

### Yeast one-hybrid assays

The yeast one-hybrid (Y1H) system consisted of a bait vector and a prey vector, both of which were constructed through Gateway Cloning Technology (Curtis and Grossniklaus 2003). For the construction of bait vectors, the *SlLCYE* promoter fragment was repeated three times and chemically synthesized, and the *SlGCR* promoter fragment was amplified by PCR, both of which were individually inserted into the pHis-Leu-GW. For prey vectors, full-length CDS of *SlGCR* and *SlRIN* were assembled into the pDEST22 respectively, and an empty pDEST22 vector was used as a control. The recombinant bait vectors were first transformed into yeast strain AH109 cells, and the recombinant prey vectors were subsequently transformed into the cells containing the bait vectors. The yeast cells, which could grow on the SD-/Leu-/Trp-/His medium, were selected to perform a dilution assay as described previously (Ying et al. 2020).

### Statistics

Unless specifically described, the data are presented as means ± SD for three biological replicates. Unpaired two-tailed Student’s *t*-tests were used to compare individual treatments with their relevant controls. *P* < 0.05 were considered significant. GraphPad Prism 8.0 and Microsoft Excel 2019 were used for analysis.

### Accession numbers

Sequence data from this article can be found in the Solanaceae Genomics Network (SGN, https://solgenomics.net/) under the following accession numbers: *SlGCR* (*Solyc12g098370*), *SlPSY1* (*Solyc03g031860*), *SlPDS1* (*Solyc03g123760*), *SlZDS* (*Solyc01g097810*), *SlZISO* (*Solyc12g098710*), *SlCRTISO* (*Solyc10g081650*), *SlLCYE* (*Solyc12g008980*), *SlLCYB* (*Solyc06g074240*), *SlHYDB* (*Solyc04g051190*), *SlHYDE* (*Solyc10g083790*), *SlRIN* (*Solyc05g012020*), *SlUBI* (*Solyc01g056940*). RNA-Seq data from this article can be found in the Genome Sequence Archive at the Big Data Center, Beijing Institute of Genomics, Chinese Academy of Sciences, under the accession number CRA007919.

## Supplementary Information

Below is the link to the electronic supplementary material.

Supplementary file1 (DOCX 1101 KB)

Supplementary file2 (XLSX 18 KB)

Supplementary file3 (XLSX 87 KB)

## Data Availability

All data generated or analyzed during this study are included in this published article and its supplementary information files.
