# Supplementary material for: G2-LIKE CAROTENOID REGULATOR (SlGCR) is a positive regulator of lutein biosynthesis in tomato
Source: aBIOTECH. 2022 Nov 29;3(4):267–80. doi: 10.1007/s42994-022-00088-z (PMC9755792; doi:10.1007/s42994-022-00088-z)
Supplement: Supplementary file 1 — Supplementary file1 (DOCX 1101 KB) [file 42994_2022_88_MOESM1_ESM.docx]

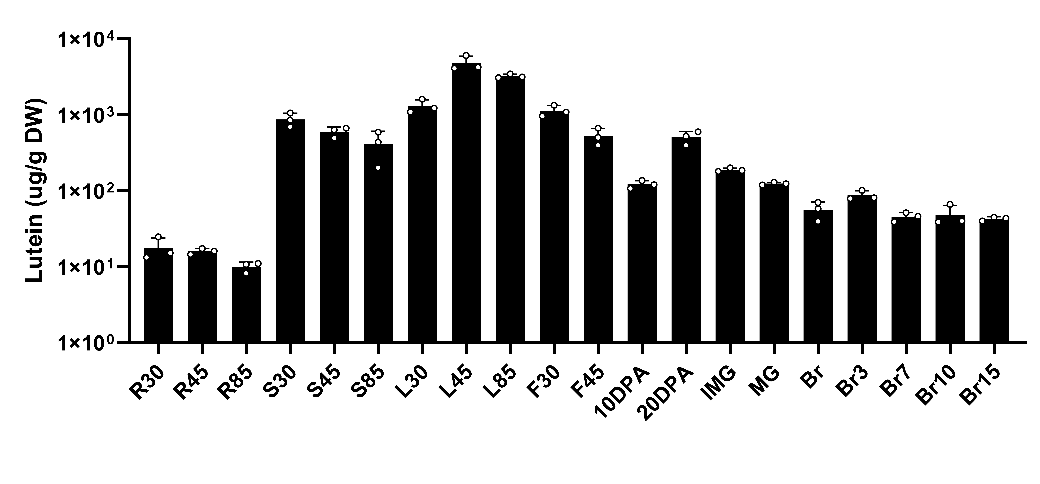


**Fig. S1 Lutein content in different tissues during tomato developmental process.** Root (R), stem (S), leaf (L), and flower (F) samples were harvested at 30 DPG, 45 DPG, 85 DPG. Fruit samples were harvested at 10 DPA, 20 DPA, immature green (IMG), mature green (MG), breaker (Br), 3 days post breaker stage (Br3), Br7, Br10, Br15. Error bars represent the SD (n=3).


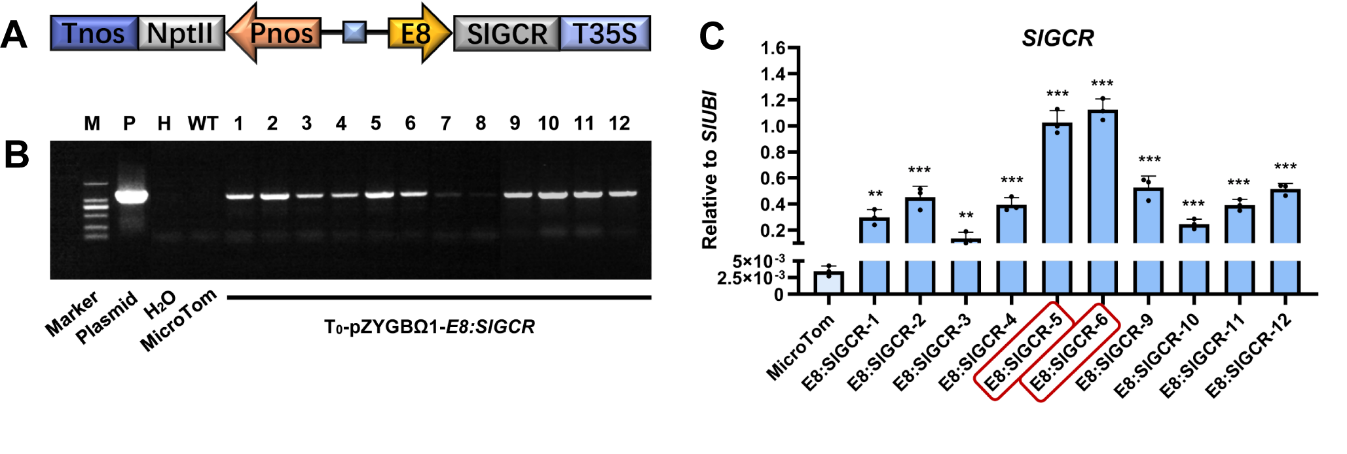


**Fig. S2 Fruit-specific overexpression lines of T_0_-generation.** A, Schematic diagram of fruit-specific overexpression vector. B, Agarose gel electrophoresis showing the identification of positive T_0_-*E8:SlGCR* plants. C, Expression levels of *SlGCR* in T_0_-*E8:GCR* fruits at the Br7 stage. Error bars represent the SD (n=3). **(*P*<0.01) and ***(*P*<0.001) compared to MicroTom at the same stage (Student’s *t*-test).


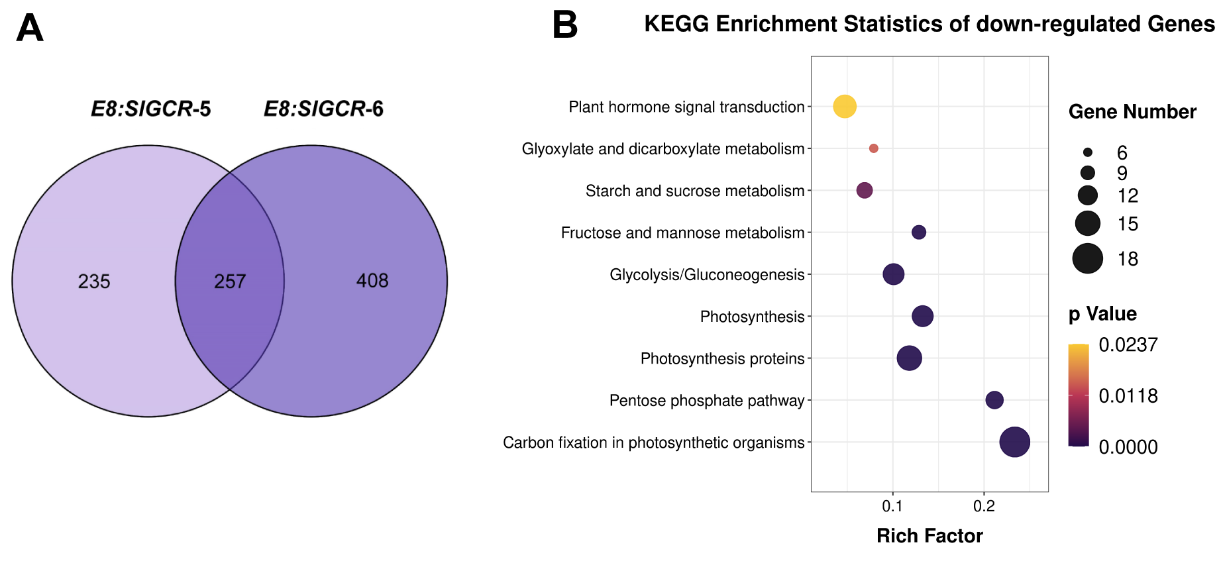


**Fig. S3 Downregulated differentially expressed genes (DEGs) shared by both lines compared with MicroTom.** A, Venn diagram showing the common and specific downregulated genes in T_1_-*E8:SlGCR*-5 and 6. B, KEGG enrichment statistics of co-downregulated genes in both T_1_-*E8:SlGCR* lines. Rich factor reflects the proportion of differentially expressed genes in a given pathway. The size of each node represents the number of enriched genes. *P* values are indicated by different colors, changing from yellow to purple.


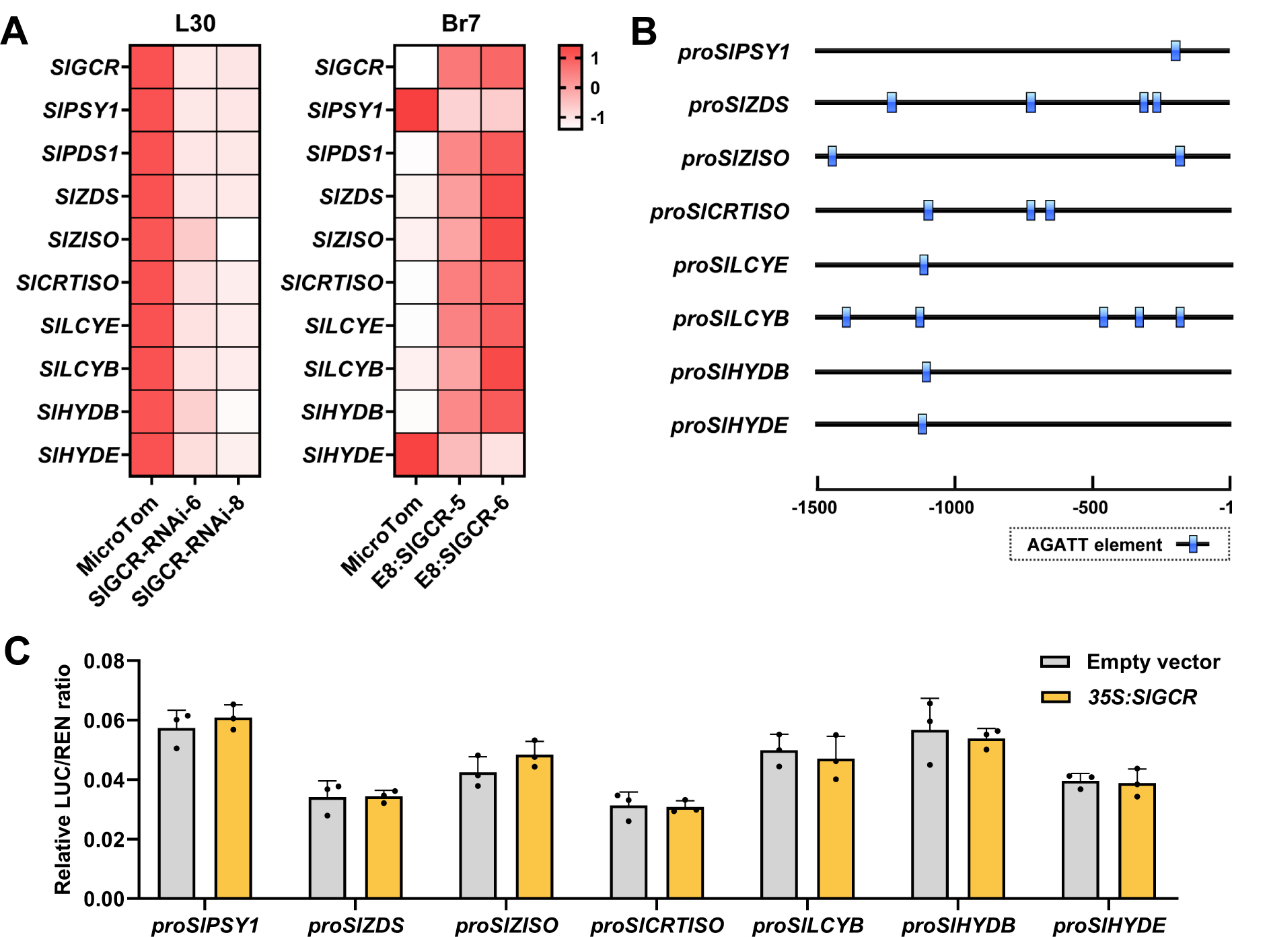


**Fig. S4 Screening for the direct target genes of SlGCR.** A, RT-qPCR showing the expression levels of carotenoid biosynthetic genes in T_1_-*SlGCR*-RNAi and T_1_-E8:*SlGCR*. Leaves at the 30 DPG stage, L30. Fruits at the Br7 stage, Br7. B, Schematic diagrams of AGATT elements on promoters of carotenoid biosynthetic genes (1500 bp upstream the ATG site) locus. C, Relative LUC/REN ratio showing no significance existing in the transcriptional activity of promoters caused by SlGCR. Error bars represent the SD (n=3).


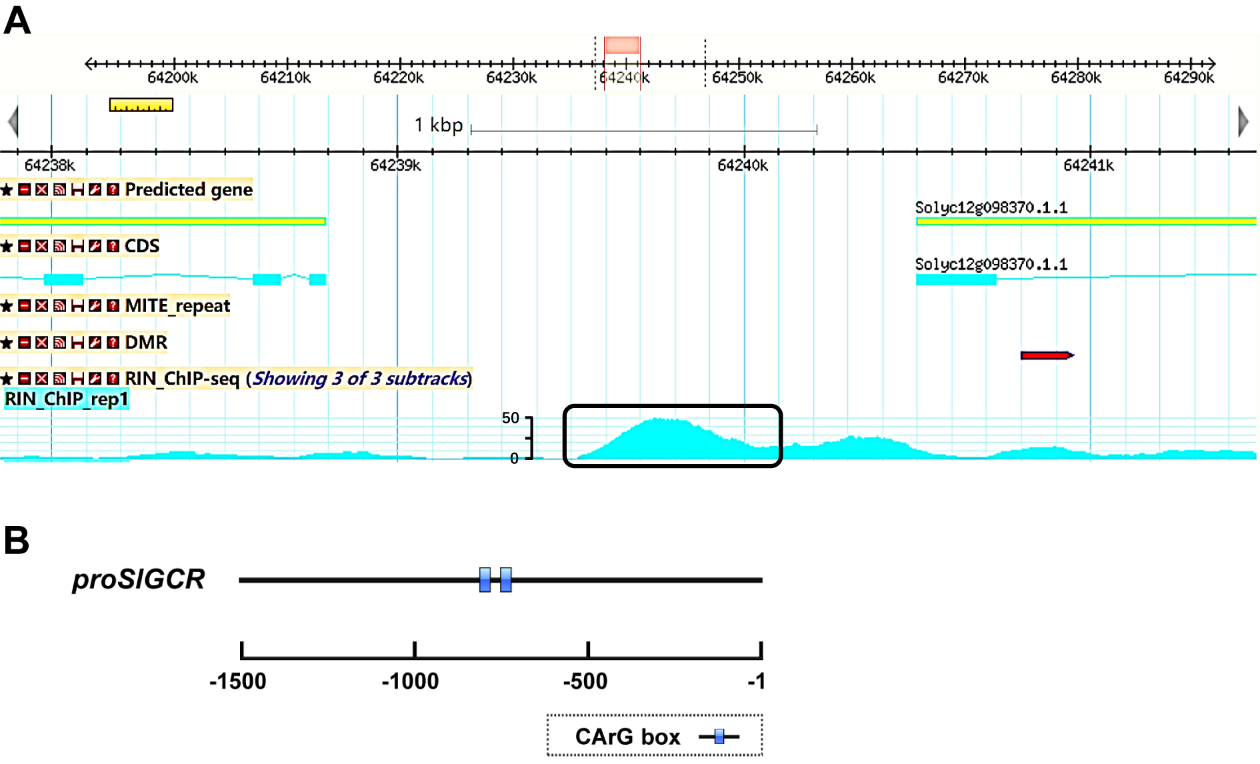


**Fig. S5 *SlGCR* promoter has the potential binding sites of SlRIN.** A, Chromatin immunoprecipitation sequencing (ChIP-seq) showing the enrichment of SlRIN on the *SlGCR* promoter (Tomato Epigenome Database, <http://ted.bti.cornell.edu/epigenome/>). B, Schematic diagram of CArG boxes on *SlGCR* promoter (1500 bp upstream of the ATG site).

| **Carotenoids (μg/g DW)** | **MicroTom** | ***SlGCR*-RNAi-5** | | ***SlGCR*-RNAi-6** | | ***SlGCR*-RNAi-8** | | ***SlGCR*-RNAi-10** | |
| --- | --- | --- | --- | --- | --- | --- | --- | --- | --- |
| γ-Carotene | 184.00±7.63 | 125.97±1.59*** | 86.30±9.58*** | | 134.03±5.53** | | 109.90±7.37*** | |  |
| α-Carotene | 154.43±10.68 | 99.93±4.73** | 73.77±4.20*** | | 108.67±6.53** | | 95.80±3.04*** | |  |
| Lutein | 7172.00±208.91 | 5834.67±244.78** | 5083.33±353.77*** | | 6281.00±194.49** | | 5294.00±125.53*** | |  |
| β-Carotene | 1173.33±50.12 | 907.73±40.44** | 741.43±69.26*** | | 1024.97±36.55* | | 864.87±42.83** | |  |
| Zeaxanthin | 77.52±6.43 | 38.56±4.98** | 10.35±4.11*** | | 47.20±3.72** | | 26.67±0.62*** | |  |

**Table S1. Contents of the major carotenoids in leaves at the 45 DPG stage.** Data are shown as means ± SD (n = 3). *(*P*<0.05), **(*P*<0.01) and ***(*P*<0.001) compared to MicroTom at the same stage (Student’s *t*-test).

| **Gene locus** | **Name** | **Primer sequence** | **Annotation** |
| --- | --- | --- | --- |
| *Solyc12g098370* | *SlGCR*-F | ATGAAAATGTACTCTTCTTTAG | Cloning |
| *Solyc12g098370* | *SlGCR*-R | TCAGGAACTAGTCATCATCC | Cloning |
| *Solyc05g012020* | *SlRIN*-F | ATGGGTAGAGGGAAAGTAG | Cloning |
| *Solyc05g012020* | *SlRIN*-R | TCAAAGCATCCATCCAGGTAC | Cloning |
| *Solyc12g098370* | q*SlGCR*-F | CTACAAGAGCAGCTAGAGGTAC | RT-qPCR |
| *Solyc12g098370* | q*SlGCR*-R | GTTCCCTAGCAGTGTCTAGATC | RT-qPCR |
| *Solyc05g012020* | q*SlRIN*-F | GCAAAGAGAAGAAATGGACTCC | RT-qPCR |
| *Solyc05g012020* | q*SlRIN*-R | GGGTTCCTTCAAGTGTACCATA | RT-qPCR |
| *Solyc03g031860* | q*SlPSY1*-F | TGAATCAAAGGCAACAACAGAG | RT-qPCR |
| *Solyc03g031860* | q*SlPSY1*-R | CTGTGCTAATTCATCTTGAGGC | RT-qPCR |
| *Solyc03g123760* | q*SlPDS1*-F | TCAAAACTCCGAGGTCTGTTTA | RT-qPCR |
| *Solyc03g123760* | q*SlPDS1*-R | GCCAAGTATTTCTGTTTCGTGT | RT-qPCR |
| *Solyc01g097810* | q*SlZDS*-F | TTGGACAATCTCCTCTATACGC | RT-qPCR |
| *Solyc01g097810* | q*SlZDS*-R | TTGATAGAGGCATGTAAGGGTC | RT-qPCR |
| *Solyc12g098710* | q*SlZISO*-F | CCTTCTTCTTCCTATACCCGTC | RT-qPCR |
| *Solyc12g098710* | q*SlZISO*-R | TGCCTGGTAATCCTCATAATCC | RT-qPCR |
| *Solyc10g081650* | q*SlCRTISO*-F | GGACTCTCTCCGAAAGACTATG | RT-qPCR |
| *Solyc10g081650* | q*SlCRTISO*-R | GTTCCCACCTCCTTAAAGAGAA | RT-qPCR |
| *Solyc12g008980* | q*SlLCYE*-F | CTTATGGAGTGGAAGTTGAGGT | RT-qPCR |
| *Solyc12g008980* | q*SlLCYE*-R | CTTCTAAAGATTGAGCGTCGTG | RT-qPCR |
| *Solyc06g074240* | q*SlLCYB*-F | TTTATAGAGTATGACAGGCC | RT-qPCR |
| *Solyc06g074240* | q*SlLCYB*-R | CAAATGAGAATCCCTCCAATCCA | RT-qPCR |
| *Solyc04g051190* | q*SlHYDB*-F | TGGAAAGATATCTCTCCGAAGC | RT-qPCR |
| *Solyc04g051190* | q*SlHYDB*-R | CTCTTCTTCGTCTACCATCCTC | RT-qPCR |
| *Solyc10g083790* | q*SlHYDE*-F | ATAGAGATCCAAGCATCCTTCG | RT-qPCR |
| *Solyc10g083790* | q*SlHYDE*-R | AAACTGAACCTGTGGTTTCATG | RT-qPCR |
| *Solyc01g056940* | q*SlUBI*-F | GCCAAAGAAGATCAAGCACA | RT-qPCR |
| *Solyc01g056940* | q*SlUBI*-R | TCAGCATTAGGGCACTCCTT | RT-qPCR |

**Table S2. Primers used in this study.**
